# Improving efficiency of on-site monitoring in multicentre clinical trials by targeting visits

**DOI:** 10.1186/1745-6215-16-S2-O49

**Published:** 2015-11-16

**Authors:** Carol Knott, Elsa Valdes-Marquez, Martin Landray, Jane Armitage, Jemma Hopewell

**Affiliations:** 1 Clinical Trial Service Unit, Nuffield Department of Population Health, University of Oxford, Oxford, UK

## Background

Monitoring guidance for clinical trials recommends the use of central and on-site monitoring practices. Comparing the efficiency of on-site monitoring visits targeted as a result of central statistical monitoring (CSM) procedures, versus those that were not, could help to improve trial conduct.

## Methods

The monitoring plan in a large long-term multicentre international trial required that sites received routine monitoring visits every nine months. Oversight of this trial was augmented by central statistical monitoring that identified high scoring sites as priority for further investigation. To validate this approach high scoring sites, and some low scoring sites (in the same countries) identified by the country teams as potentially problematic were visited.

## Results

Over a twelve month period 21 sites (12 identified by CSM, 9 others as comparators) received a comprehensive monitoring visit from the senior monitor. Only 1 site identified by CSM had no findings, versus 7 of the comparator sites. Minor findings indicative of ‘sloppy practice’ were identified at 12 sites (10 CSM versus 2 comparator). At 1 site identified by CSM there were serious findings indicative of an under-performing site.

## Conclusion

On-site visits triggered by CSM are more efficient than undirected routine visits. Furthermore, information from CSM can help focus the nature of on-site visits and any interventions required to improve site quality, e.g. additional training on data collection or event reporting. Study costs will be reduced if resources are directed to improving the performance of sites of concern, leaving lower risk sites to receive less frequent on-site monitoring.

